# An Atypical Presentation of Diffuse Large B-Cell Lymphoma

**DOI:** 10.7759/cureus.12527

**Published:** 2021-01-06

**Authors:** Sofia Garcês Soares, Teresa Martins Mendes, Catarina Couto, Diana Pereira Anjos, Ana João Sá

**Affiliations:** 1 Internal Medicine, Centro Hospitalar Tâmega e Sousa, Penafiel, PRT

**Keywords:** non hodgkin's lymphoma, adrenal glands, primary adrenal insufficiency

## Abstract

Diffuse large B-cell lymphoma (DLBCL) is the most common type of non-Hodgkin's lymphoma (NHL) and it can metastasize to extranodal sites. The involvement of the adrenal glands is rare. In this report, we discuss the case of a 65-year-old man with complaints of asthenia, anorexia, hypersudoresis, and a weight loss of 10 kg in the month prior to his presentation. Suprarenal insufficiency and bilateral masses with heterogeneous contrast uptake in the adrenal glands were documented on a thoracoabdominopelvic CT. Infectious causes and functioning tumors were excluded. After an exhaustive study, DLBCL was diagnosed. Through this case report, the authors intend to sound the alert on the existence of a rare presentation of DLBCL.

## Introduction

Primary non-Hodgkin's lymphomas (NHL) are more common in males than females and the average age at diagnosis is 68 years. Diffuse large B-cell lymphoma (DLBCL) is the most common type of NHL, accounting for 30-40% of cases, and it often presents with nonspecific clinical features. The most common signs and symptoms are fever, abdominal pain, low back pain, and weight loss. In about 40% of cases, DLBCL can metastasize to extranodal sites. The endocrine system is affected in 3% of cases and the involvement of the adrenal glands is rare [1-5].

## Case presentation

**Figure 1:**
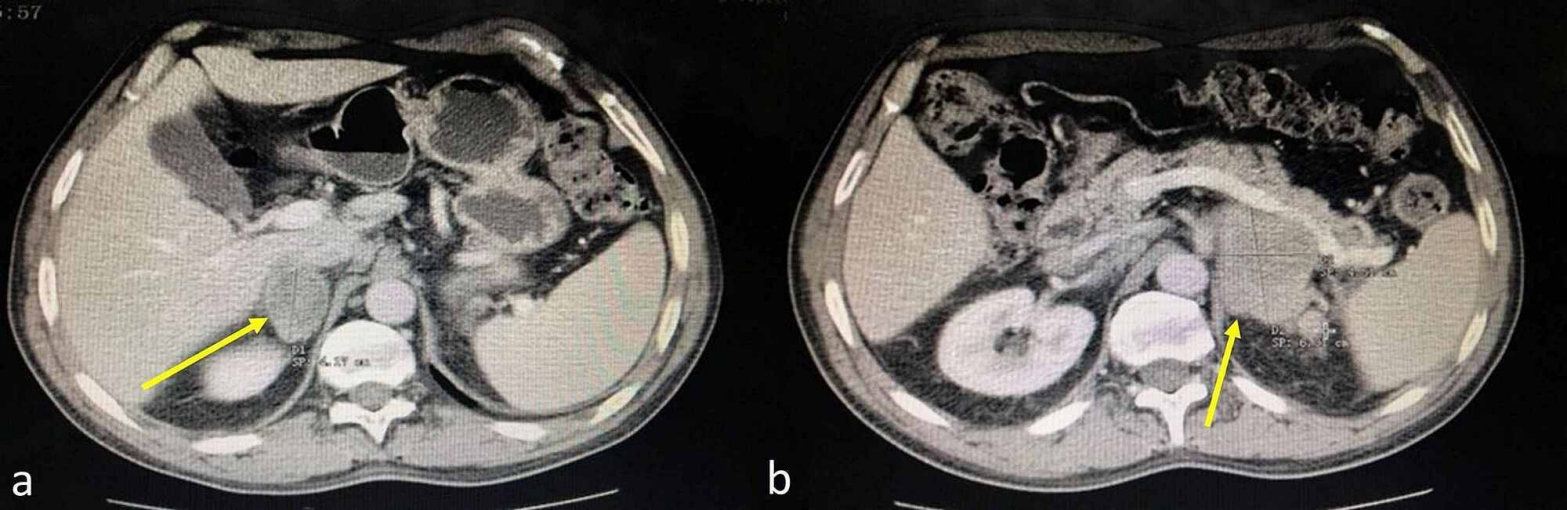
Masses of adrenal glands on thoracoabdominopelvic CT (arrows) CT: computed tomography

A 65-year-old man, independent in all activities of daily living, with a personal history of bilateral hydrocelectomy, active smoking, arterial hypertension, and dyslipidemia, who had been regularly treated with valsartan 80 mg id and pitavastatin 2 mg id respectively, was referred to the Emergency Department due to asthenia, anorexia, hypersudoresis, and weight loss of 10 kg in the previous month. Upon admission, he had no relevant changes on physical examination. Initial studies demonstrated normochromic normocytic anemia (hemoglobin of 9.9 g/dL), Na^+ ^of 136 mmol/L (136-144), K^+ ^of^ ^5.2 mmol/L (3.5-5.1), C-reactive protein (CRP) of 131.9 mg/L (<7.5), and lactic dehydrogenase (LDH) of >800 UI/L (266-500). A thoracoabdominopelvic axial CT revealed bilateral masses with heterogeneous contrast uptake in the adrenal glands (the largest being 6 x 5 cm, lobulated, poorly delimited, and infiltrative), suggestive of metastases (Figure 1), and retroperitoneal adenomegalies in left peri-aortic and para-aortic topography at the level of the renal hilum, the most prominent being 1.7 x 1.2 cm.

The patient was admitted to the Internal Medicine ward in order to study the bilateral lesions of the adrenal glands, where he would later develop a fever (maximum axillary temperature of 38.6 ºC), hypotension (mean arterial pressure of 77 mmHg) and hyponatremia (Na^+^ of^ ^131 mmol/L).

Infectious causes were excluded based on the following findings: negative HIV; negative cultural exams; negative *Mycobacterium tuberculosis* DNA in urine; bronchoalveolar lavage (BAL) with negative testing for acid-resistant bacilli and mycobacteriological exam and transthoracic echocardiogram without evidence of valve vegetation.

However, after performing a dosing of morning cortisol and adrenocorticotropic hormone (ACTH), which revealed values of 13.19 μg/dL (6.7-22.6) and 32.5 pg/mL (9-52) respectively, and a tetracosactide stimulation test, primary adrenal insufficiency was diagnosed. In order to investigate the cause of the adrenal insufficiency, a functioning tumor (Table 1) and also additional neoplastic causes were excluded.

**Table 1:** Summary of the study carried out to exclude a functioning tumor

Parameters	Results	Laboratory reference values
Active renin	88.3 pg/ml	4.2–45.6 pg/mL (standing), 3–28 pg/mL (lying down)
Serum aldosterone	90 pg/ml	97–626 pg/mL (standing), 42–202 pg/mL (lying down)
Plasma metanephrines	110 pmol/L	<456.3 pmol/L
Plasma normetanephrine	310 pmol/L	<982.8 pmol/L
Urinary adrenaline	<5.46 mmol/24 h	0–109 mmol/24 h
Urinary norepinephrine	267 mmol/24 h	8–473 mmol/24 h
Urinary dopamine	2,019 mmol/24 h	424–2,612 mmol/24 h
Urinary metanephrine	900 mmol/24 h	264–1,729 mmol/24 h
Urinary normetanephrine	2,529 mmol/24 h	480–2,424 mmol/24 h

In the process of excluding other neoplastic causes, PSA dosing was performed and it was found to be normal (0.31 ng/mL; 0.0-4.00). A proteinogram showed no monoclonal peaks and peripheral blood immunophenotyping showed no evidence of monoclonal cells. When brochfibroscopy was performed, there were no endoscopic signs of tumor pathology either; BAL cytology did not show any malignant cells and its immunophenotyping did not show any monoclonal cells. An aspiration biopsy of an adenomegaly next to an adrenal gland was also performed and, despite being suggestive of a malignant neoplasm, it was not conclusive.

**Figure 2:**
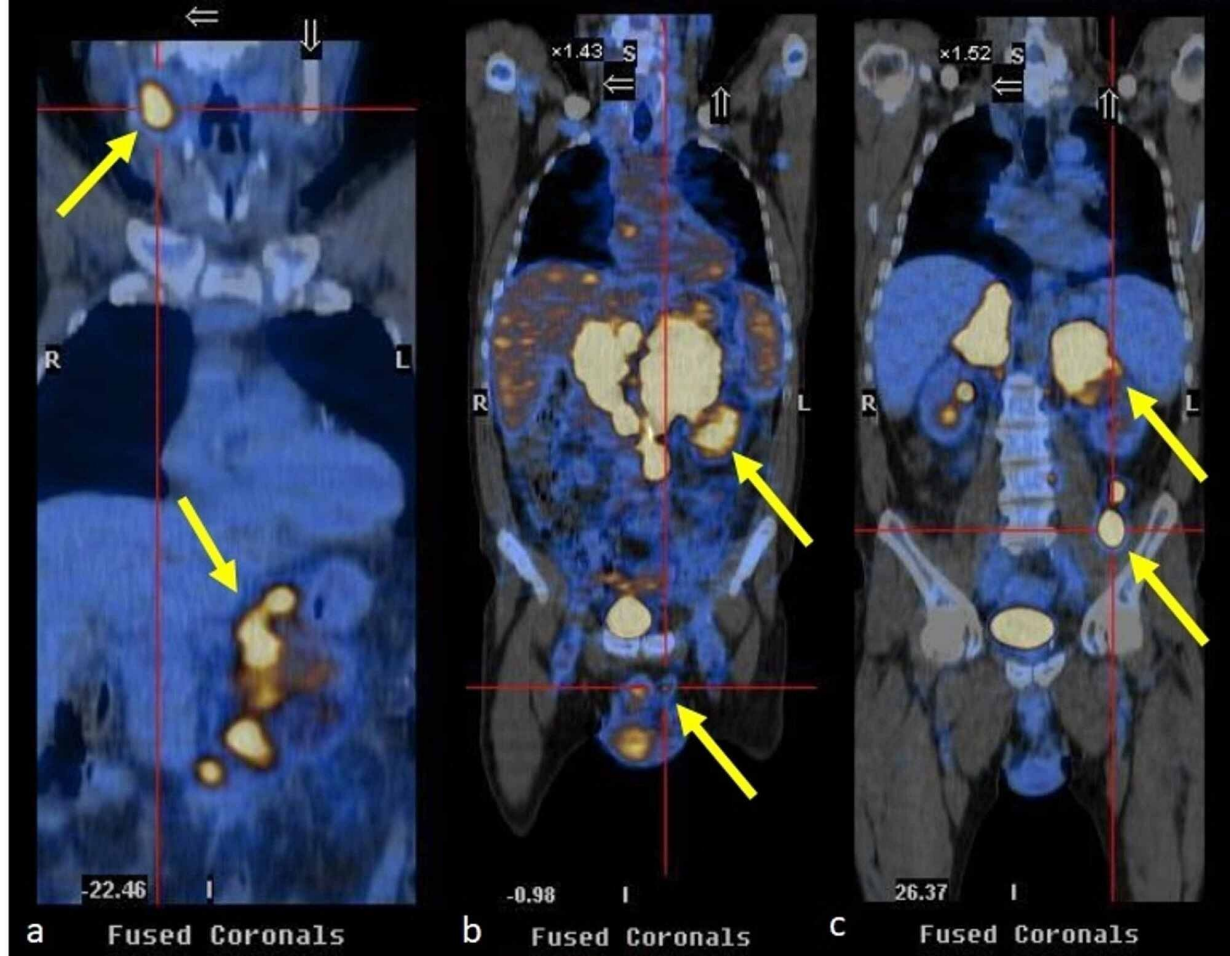
Positron emission tomography showing foci suggesting malignancy

It was then decided to carry out a positron emission tomography (PET), which demonstrated foci suggesting malignancy in the oropharynx; cervical, left axillary, abdominal ganglia; adrenal glands, spermatic cords, and posterior area of ​​the right thigh (Figure 2).

An upper gastrointestinal endoscopy was performed, which revealed a thickened pre-pyloric fold and an eventual ectopic pancreas that were submitted to biopsy, revealing the involvement of the gastric body by peripheral B lymphoma with DLBCL characteristics. Lower gastrointestinal endoscopy did not show any lesions suggestive of malignancy. A bone biopsy excluded spinal cord involvement.

With the diagnosis of DLBCL with extranodal involvement, the patient was transferred to the Hematology department of a central hospital, where he underwent six cycles of chemotherapy with R-CHOP (rituximab, cyclophosphamide, doxorubicin, vincristine, prednisolone) and three cycles of high-dose methotrexate with complete remission until to date; he has been maintaining follow-up appointments in this specialty.

## Discussion

The most likely causes of adrenal masses are metastatic lesions (lung, breast, stomach, kidney, melanomas, and lymphomas), congenital hyperplasia, bilateral adenoma, infiltrative disease, and tuberculosis [1-6].

In this clinical case, given the presence of fever, infectious causes were initially excluded. Additionally, and as a fundamental step in the first approach to adrenal masses, a functioning tumor was excluded. To achieve this, a thorough clinical evaluation of the signs and symptoms suggestive of a functioning tumor (hypercortisolism, hyperaldosteronism, and pheochromocytoma) confirmed by biochemical tests should be made. Signs and symptoms such as central obesity, arterial hypertension, fatigue, bruises, violet streaks, and hirsutism are suggestive of hypercortisolism, while arterial hypertension, edema, and hypokalemia are suggestive of hyperaldosteronism; severe headache, weight loss, profuse sweating, palpitations, and arterial hypertension are suggestive of pheochromocytoma. As previously described, this patient, despite having nonspecific complaints, had no relevant changes on physical examination when he was first admitted. Additionally, dosing of morning cortisol, ACTH, plasma aldosterone ratio/plasma renin activity, and metanephrine/catecholamine assay in 24-hour urine also showed no relevant changes.

In view of bilateral adrenal involvement, in the presence or absence of a low tension profile of ionic changes, adrenal insufficiency should also be screened by tetracosactide stimulation test. It is very important to be mindful of this, as it can save some time and prevent further deterioration like in this case. Although this patient did not present initially with a low tension profile or hyponatremia, a tetracosactide stimulation test was performed and adrenal insufficiency was diagnosed. Under a hydrocortisone dose of 15 mg/day, normalization of the tension profile and ionogram was verified.

Imaging characteristics such as a dimension greater than 4 cm, heterogeneous texture, irregular shape (all features present in this case), and hypervascularization suggest malignancy. The nonspecificity of this clinical case associated with bilateral adrenal masses and glandular insufficiency led to an aspiration biopsy of an adenomegaly close to one of the adrenal glands, the histology of which, although suggestive of neoplasia, was not conclusive, leading to a delay in the diagnosis. PET is based on the principle of increased glucose metabolism in malignant lesions, with a sensitivity of 100% and specificity of 80-100% in the diagnosis of adrenal masses [1]. This exam was very useful because it identified anatomical sites that had foci of malignancy more accurately. Still, as part of the investigation of a neoplastic cause, a gastric biopsy was performed, which verified the diagnosis of DLBCL.

The primary lymphoma involvement of the adrenal glands is rare and, in 50-70% of the cases, it has a bilateral presentation. Secondary involvement has been reported in about 25% of autopsies and is typically unilateral [1-7].

Treatment of DLBCL includes surgery, radiation, and chemotherapy [1,3]. The systemic chemotherapy regimen R-CHOP is the first-line treatment because although these lymphomas can be located, they can relapse in a large number of cases [2,3]. Bilaterality and the presence of adrenal insufficiency, secondary to tumor infiltration, are considered factors of poor prognosis. The prognosis is reserved, with a survival rate of 17.5% over a period of one year [1-3,7-8].

## Conclusions

In this report, we discussed a case of bilateral NHL of the adrenal glands, whose diagnostic hypothesis, although rare, should always be considered during the diagnosis process of masses of the adrenal gland, especially when the involvement is bilateral and when there is adrenal insufficiency. The nonspecificity of the symptoms underlying the adrenal insufficiency and its similarity with the symptoms that may be inherent to the neoplasia itself make it difficult to recognize it.

In this clinical case, despite the bilaterality of the adrenal gland lesions, it was not possible to conclude whether these lesions were primary or secondary. As described, the PET performed ultimately revealed several foci suggestive of malignancy.
